# High-throughput bone and cartilage micropellet manufacture, followed by assembly of micropellets into biphasic osteochondral tissue

**DOI:** 10.1007/s00441-015-2159-y

**Published:** 2015-04-30

**Authors:** Betul Kul Babur, Kathryn Futrega, William B. Lott, Travis Jacob Klein, Justin Cooper-White, Michael Robert Doran

**Affiliations:** Stem Cell Therapies Laboratory, Institute of Health and Biomedical Innovation, Queensland University of Technology at the Translational Research Institute, Brisbane, Australia; Cartilage Regeneration Laboratory, Institute of Health and Biomedical Innovation, Queensland University of Technology, Brisbane, Australia; Tissue Engineering and Microfluidics Laboratory, Australian Institute for Bioengineering and Nanotechnology, The University of Queensland, Brisbane, Australia; Mater Medical Research - University of Queensland, Brisbane, Australia

**Keywords:** Articular cartilage, Tissue engineering, Osteochondral defect repair, Mesenchymal stem/stromal cells, Micropellets, Induction media, Human

## Abstract

Engineered biphasic osteochondral tissues may have utility in cartilage defect repair. As bone-marrow-derived mesenchymal stem/stromal cells (MSC) have the capacity to make both bone-like and cartilage-like tissues, they are an ideal cell population for use in the manufacture of osteochondral tissues. Effective differentiation of MSC to bone-like and cartilage-like tissues requires two unique medium formulations and this presents a challenge both in achieving initial MSC differentiation and in maintaining tissue stability when the unified osteochondral tissue is subsequently cultured in a single medium formulation. In this proof-of-principle study, we used an in-house fabricated microwell platform to manufacture thousands of micropellets formed from 166 MSC each. We then characterized the development of bone-like and cartilage-like tissue formation in the micropellets maintained for 8–14 days in sequential combinations of osteogenic or chondrogenic induction medium. When bone-like or cartilage-like micropellets were induced for only 8 days, they displayed significant phenotypic changes when the osteogenic or chondrogenic induction medium, respectively, was swapped. Based on these data, we developed an extended 14-day protocol for the pre-culture of bone-like and cartilage-like micropellets in their respective induction medium. Unified osteochondral tissues were formed by layering 12,000 osteogenic micropellets and 12,000 chondrogenic micropellets into a biphasic structure and then further culture in chondrogenic induction medium. The assembled tissue was cultured for a further 8 days and characterized via histology. The micropellets had amalgamated into a continuous structure with distinctive bone-like and cartilage-like regions. This proof-of-concept study demonstrates the feasibility of micropellet assembly for the formation of osteochondral-like tissues for possible use in osteochondral defect repair.

**Electronic supplementary material:**

The online version of this article (doi:10.1007/s00441-015-2159-y) contains supplementary material, which is available to authorized users.

## Introduction

Articular cartilage has limited intrinsic regeneration capacity. The degeneration of articular cartilage leads to osteoarthritis (OA), which is the most common form of joint disease and one of the leading causes of disability worldwide (Brand et al. 2014; Chen et al. 2012; Centers for Disease Control and Prevention 2013; Woolf and Pfleger 2003). The capacity to repair acute cartilage defects effectively could delay or prevent the development of OA in many patients. A number of strategies for the repair of acute cartilage defects have been proposed which and these range from the filling of the defect with cells, with natural/synthetic hydrogels/scaffolds, or with combinations of both (Behrens et al. 2006; Brittberg et al. 1994; Libera et al. 2006). A number of challenges are associated with any cartilage defect repair and perhaps the most significant of these is the effective integration of the repair tissue with the adjacent native tissue (Archer et al. 2006; Fisher et al. 2014). Because chondrocyte cell density is low in cartilage tissue, and these cells have a limited capacity to migrate through cartilage matrix at wound edges, the borders surrounding the repair tissue are not iteratively remodeled and  repair tissue is not easily integrated with the adjacent native tissue. Additionally, the underlying bone often incurs simultaneous damage during the initial injury and the building of new tissue on this damaged foundation can be problematic (Muehleman et al. 2009). In procedures such as mosaicplasty, the underlying bone and associated cartilage are harvested from a donor location and transplanted into a critical defect site (Matsusue et al. 1993). The inclusion of the bone layer is essential as it facilitates tissue integration and provides a foundation for the donor cartilage tissue (Huntley et al. 2005). Whereas the bone layer rapidly and iteratively remodels, the integration of the cartilage layer can be delayed or even remain incomplete. A ring of dead tissue can often be observed around the perimeter of a mosaicplasty tissue plug (Huntley et al. 2005). Nevertheless, the inclusion of the bone layer does indeed enhance integration and the probability of a successful repair. A fundamental drawback in mosaicplasty is the limited supply of autologous donor tissue available for harvest and the associated damage at the donor site. These limitations are driving the development of tissue engineering solutions that will enable the manufacture of either synthetic or biosynthetic osteochondral tissues.

Approaches trialed in the manufacture of artificial osteochondral tissues include the use of bilayer scaffolds, separate scaffolds for bony and chondral layers, scaffolds for bony layer/scaffold-free for chondral layers and single homogeneous or heterogeneous scaffolds (Nooeaid et al. 2012). Cell-free studies have also utilized scaffolds to fill the defect site, with the anticipation that biological “cues” incorporated into the scaffold will direct host cells to repopulate the scaffold in a useful and organized manner (Filardo et al. 2013; Filová et al. 2013; Gotterbarm et al. 2006). This cell-free approach is probably not suitable for large chondral defects for reasons similar to those for the failure of mosaicplasty plugs to integrate; because chondrocytes have a limited capacity to migrate from the dense matrix in the adjacent native tissue, the probability that cells would be able to populate a large scaffold is low. For this reason, cell-free studies are often coupled with the microfracture technique, which stimulates bleeding and recruits cells from within the underlying bone marrow (Erggelet et al. 2009; Gille et al. 2010; Siclari et al. 2012). Additionally, most scaffold approaches aim to use the scaffolds as a temporary extracellular matrix (ECM) support and assume that the biodegradation of the scaffold will be synchronized with the de novo tissue formation. This synchronization is technically challenging when the scaffold has multiple phases or components. Other studies have explored the repair of full-thickness osteochondral defects with scaffold-free approaches. However, in such cases, the repair tissue only includes a cartilage phase (Cheuk et al. 2011; Brehm et al. 2006) and the de novo tissue relies on the existing bone to function as both the tissue foundation and interface.

Bone marrow-derived mesenchymal stem/stromal cells (MSC) are frequently utilized in osteochondral defect studies (Chen et al. 2013a, 2013b; Loken et al. 2008; Liu et al. 2006), because these cells are assumed to have the potential to differentiate into either chondrocyte- or osteoblast-like cells. The chondrogenic or osteogenic phenotype derived from MSC populations is highly sensitive to the medium formulation and chondrogenic and osteogenic media traditionally have different formulations from each other (Pittenger et al. 1999). Construction of a continuous osteochondral tissue in vitro presents a real challenge as it obligates the culture of both tissue types in a single medium cocktail. Some groups have attempted to overcome this challenge by culturing composite tissues in custom-made double-chamber bioreactors that aim physically to isolate each tissue type in their unique medium formulations (Liu and Jiang 2013; Malafaya and Reis 2009). We reason that a more rational approach might be to manufacture MSC micropellets, to induce chondrogenic or osteogenic differentiation in discrete culture systems and then to assemble the partially mature micropellets into a biphasic tissue in a common culture. Based on this rationale, we describe a method for the high-throughput manufacture of discrete bone-like and cartilage-like micropellets with the subsequent assembly of the micropellets into a biphasic osteochondral-like tissue.

Bone-like and cartilage-like micropellets were manufactured by using a modified microwell platform previously described by our group (Markway et al. 2010; Kabiri et al. 2012). Using this modified method, we systematically characterized the phenotypic change of bone marrow-derived MSC micropellets exposed sequentially to osteogenic and/or chondrogenic medium. Following the characterization and optimization of this process, we assembled bone-like and cartilage-like micropellets into an osteochondral tissue and cultured this construct in a single medium formulation.

## Materials and methods

### Experimental design

This study has two parts. In the first part, the phenotypic characteristics of micropellets formed from human bone marrow-derived MSC cultured for 14 days under four different sequential medium conditions are characterized. The medium conditions are described in Table 1 and defined by the following abbreviations: CC (chondrogenic medium for 14 days), CO (chondrogenic medium for the first 8 days and osteogenic medium for the last 6 days), OC (osteogenic medium for the first 8 days and chondrogenic medium for the last 6 days), OO (osteogenic medium for 14 days). After a 14-day exposure to osteogenic medium and/or chondrogenic medium, micropellets were assessed for DNA, chondrogenic and osteogenic differentiation, histological features and gene expression. In the second part of the study, chondrogenic and osteogenic micropellets are assembled at various time points to form a continuous biphasic osteochondral-like tissue. The biphasic tissue was assessed for cartilage and bone histological features.

**Table 1:** Culture conditions defined according to media type and culture duration: the first letter “C” or “O” indicates the culture medium formulation over the first 8 days of culture and the second letter “C” or “O” indicates the culture medium formulation over the subsequent 6 days of culture. The media types were swapped at day 8 and not at day 7, because the media were changed every second day throughout the culture period

Abbreviation	Medium for first 8 days	Medium for last 6 days
CC	Chondrogenic	Chondrogenic
CO	Chondrogenic	Osteogenic
OC	Osteogenic	Chondrogenic
OO	Osteogenic	Osteogenic

### Microwell fabrication and surface modification

We used an in-house fabricated microwell surface in order to manufacture micropellets efficiently (Babur et al. 2013). In brief, a polystyrene microwell mold having an array of microwells (each with dimensions of 360 × 360 × 180 μm and 600 microwells/cm^2^) was used to generate sheets of polydimethylsiloxane (PDMS, Slygard) with microwells. The process of fabricating the mold is described in detail elsewhere (Babur et al. 2013). Discs of 2 or 10 cm^2^ were punched from the sheets of PDMS and used to make multiwell inserts with 1200 and 6000 microwells, respectively. The PDMS discs were glued (Silicone Glue) into 24-well plates and 6-well plates (Nunc), respectively. The plates with the microwell inserts were sterilized in 70 % ethanol for 1 h and then rinsed with sterile phosphate-buffered saline (PBS, Invitrogen). To prevent cell attachment, the surface were soaked with a 5 % pluronic F-127 (Sigma) solution, for 5 min and then rinsed with sterile PBS twice before cells were seeded.

### Human bone marrow MSC isolation and expansion

Bone marrow aspirates were obtained from the iliac crest of volunteer donors with written consent. This documentation is held by the Ethics Committee and all tissue samples were provided to the research team in a de-identified manner. Mater Health Services approved this consent procedure. Ethical approval for these studies was granted by the Mater Health Services Human Research Ethics Committee (Ethics number: 1541A) and the Queensland University of Technology Human Research Ethics Committee in accordance with the Australian National Statement on Ethical Conduct in Human Research.

The bone marrow aspirates were processed as described previously (Markway et al. 2010). Aspirates (∼20 ml) were diluted 1:1 by using sterile PBS and 12 ml Ficoll Paque Plus (GE Healthcare) was underlayed beneath the diluted aspirates. The Ficoll and bone marrow mixture was centrifuged at 535*g* for 20 min. The cells accumulated at the interface of the Ficoll and bone marrow were aspirated carefully and washed with sterile PBS. The cells were resuspended in low glucose Dulbecco’s modified Eagle’s medium (LG DMEM, Invitrogen) with 100 U/ml penicillin and 100 μg/ml streptomycin (PS, Invitrogen) and 10 % fetal bovine serum (FBS, Invitrogen), seeded in T175 flasks (Nunc) and incubated for 48 h before removal of the non-adherent cells. The plastic adherent cells (MSC) were cultured until confluent with media changes every 3–4 days, passaged at a 1:4 ratio three times (until passage 3) and then used in micropellet studies. MSC were expanded at 37 °C in a hypoxic incubator (2 % O_2_ and 5 % CO_2_ atmosphere) as this had been previously demonstrated to enhance proliferation and differentiation capacity (Basciano et al. 2011; Abdollahi et al. 2011). Passage-2 expanded cells were characterized for their expression of CD marker profiles associated with MSC (Dominici et al. 2006). Specifically, we confirmed that the cell populations used were CD90, CD73, CD105, CD44 and CD146 positive and CD45, CD34 and HLA-DR negative. Antibodies were purchased from Miltenyi Biotech and used as given in the manufacturer’s instructions. Stained cells were analyzed on a BD LSR-II flow cytometer.

### Micropellet formation

Micropellets were formed in the multi-well plates containing microwell inserts. In the first part of the study, 24-well plates containing 2 cm^2^ microwell inserts, each containing 1200 microwells, were used. For each sample, 1 ml medium containing 2 × 10^5^ human MSC was dispensed to each well (166 cells/microwell) and allowed to settle for micropellet formation inside the incubator. In the second part of the study, 6-well plates containing 10 cm^2^ microwell inserts, each containing 6000 microwells, were used. An aliquot of 5 ml medium containing 1 million human MSC was dispensed to each well (166 cells/microwell) and then the plates were transferred into the incubator. Micropellet formation was confirmed via microscopy the following day.

### Chondrogenic and osteogenic differentiation

Chondrogenic medium contained high glucose (HG) DMEM with 110 μg/ml sodium pyruvate (Invitrogen), PS, 10^−7^ M dexamethasone (Sigma), 200 μM ascorbic acid 2-phosphate (Sigma), 40 μg/ml L-proline (Sigma), 1 % insulin-transferrin-selenium-ethanolamine (ITS-X, Invitrogen) and 10 ng/ml recombinant human transforming growth factor-β1 (TGF-β1, Peprotech). Cultures containing chondrogenic medium were incubated in a 2 % O_2_, 5 % CO_2_ atmosphere at 37 °C.

Osteogenic medium contained HG DMEM (Invitrogen), PS, 10 % FBS, 10^−7^ M dexamethasone, 50 μM ascorbic acid 2-phosphate and 10 mM β-glycerol phosphate (Sigma). Cultures containing osteogenic medium were incubated in a 20 % O_2_, 5 % CO_2_ atmosphere at 37 °C.

In the first part of the study, 75 % of the medium was exchanged every second day for 14 days except at day 8. At day 8, 100 % of the medium was exchanged in all samples. This was because at day 8 the medium type was switched (i.e., osteogenic to chondrogenic) and the cultures were placed into different incubators at different oxygen concentrations appropriate for the medium type and corresponding differentiation process.

### Biphasic tissue assembly

In the second part of the study, the MSC micropellets were differentiated in chondrogenic medium and osteogenic medium separately and then assembled at various time points to form a continuous osteochondral-like tissue. Every second day until day 8, 75 % of the chondrogenic medium was exchanged and the chondrogenic micropellets were assembled to form a macroscopic chondrogenic tissue on day 8. Similarly, 75 % of the osteogenic medium was exchanged every second day until day 14 and then the osteogenic micropellets were assembled on top of the macroscopic chondrogenic tissue to form a biphasic tissue. The assembly of micropellets into larger tissues was achieved by using a customized PDMS funnel and layering cylinder (see Figure S2). This device was formed by using the tip of a 10 ml syringe (Terumo) inside a 6-well plate as a mold to cast the PDMS. The tip of the syringe was cut and glued to the middle of the well, the outer space left between the syringe and the well was filled with PDMS and cured and then the extra PDMS around the edges of the mold was removed to accommodate more medium in the same well. The mold was sterilized in 70 % ethanol for 1 h and then rinsed repeatedly with sterile PBS prior to use for biphasic tissue formation. On day 8, chondrogenic micropellets (12,000) were concentrated in 2 ml medium and then transferred into the funnel mold in two steps (1 ml each time, followed by 300*g* centrifugation for 2 min and removal of excess medium). The accumulation of micropellets at the bottom of the funnel mold was visually confirmed and the remaining portion of the well was then filled with 5 ml chondrogenic medium. Following the first assembly step, the assembled tissue was cultured in a 20 % O_2_, 5 % CO_2_ atmosphere at 37 °C with 75 % of the medium volume being exchanged daily. On day 14, osteogenic micropellets (12,000) were transferred into the funnel mold (by using the above approach) in order to generate a layer of bone tissue. Following the addition of osteogenic micropellets, the amalgamating tissue was cultured in chondrogenic medium for 2 more days in the funnel mold and then gently relocated into a 6-well plate. This 6-well plate was coated with PDMS and treated with 5 % pluronic F-127 to prevent cell attachment to the underlying tissue-culture-treated polystyrene but otherwise had a standard geometry. The biphasic tissue was cultured for a further 6 days in chondrogenic medium and then characterized via histology.

### Chondrogenic and osteogenic analyses

Each sample for the first part of the study consisted of 2 × 10^5^ cells (initially) in the form of 1200 micropellets. DNA, sulfated glycosaminoglycan (sGAG), calcium, alkaline phosphatase (ALP) activity and gene expression quantifications were performed on ∼1200 micropellets to obtain a single measurement. sGAG quantification in medium was performed on 0.75 ml medium collected after each medium change on every second day.

For DNA quantification, the micropellets were digested in 0.25 mg/ml papain (Sigma) solution at 60 °C overnight. DNA was quantified by using PicoGreen dsDNA Reagent and Kit (Invitrogen) according to the manufacturer’s protocol. The signal was measured by using a fluorescent plate reader (BMG Labtech) with excitation set at 480 nm and emission at 520 nm.

The quantity of sGAG in the micropellets was measured from the papain digests and sGAG in medium was measured from the medium collected during culture. The medium or digest was mixed with 1,9 dimethyl methylene blue zinc chloride double salt (DMB, Sigma) and the color change was measured by using a plate reader (Thermo Fischer) at 590 nm. Shark chondroitin sulfate (Sigma) was used to generate a standard curve.

Calcium quantification of the micropellets was achieved by incubating the tissues overnight in a tube shaker (Eppendorf) at 900 rpm in 10 % acetic acid (RCI Labscan) at room temperature. A 1 mg/ml o-Cresolphthalein Complexone (OCPC, Sigma) solution was combined with 14.8-M ethanolamine-boric acid buffer (pH 11, Sigma) and 8-hydroxyquinoline (50 mg/ml in 95 % ethanol, Merck) at a ratio of 5:5:2 and then diluted 10× with double-distilled (dd) H_2_O. The samples were mixed with OCPC solution, incubated at room temperature for 10 min and measured by using a plate reader at 575 nm. A standard curve was generated by using serial dilutions of pure CaCl_2_ (Sigma).

ALP activity was quantified in the micropellets by first lysing them in a 0.1 % Triton X-100 (Sigma) in TRIS buffer solution (pH 10.1, Sigma) contained in a tube that was then placed in a shaker at 900 rpm for 1 h at room temperature. The recovered lysate was mixed with substrate solution (1 mg/ml P-nitrophenyl phosphate disodium salt, Sigma) and incubated at room temperature for 30 min in the dark. The absorption of the incubated solution was measured in a plate reader at 405 nm.

### Gene expression analyses

RNA was extracted from micropellets by using Trizol (Invitrogen) as in the manufacturer’s suggested protocol. The isolated RNA was quantified with Nanodrop 1000 (Thermo Scientific). Reverse transcription was performed with a SuperScript III RT and oligo(dT)20 kit (Invitrogen) as in the manufacturer’s protocol to obtain complementary DNA (cDNA). A Platinum SYBR Green qPCR SuperMix-UDG kit (Invitrogen) was used to perform quantitative polymerase chain reaction (qPCR). The primer sequences (5′ to 3′, Geneworks) in Table S1 were used to perform the qPCR. The SYBR Green master mix and cDNA were combined in a 384-well plate (Applied Biosystems) by using liquid handler (epMotion M 5073, Eppendorf). The qPCR was initiated with a 2-min hold at 50 °C, continued with a 3-min hold at 95 °C and then continued with 40 cycles of 95 °C for 15 s and 60 °C for 30 s. This reaction was completed in a ViiA real time PCR machine (Applied Biosystems). The gene expression was quantified by using the ΔCt method and the relative gene expression was calculated by normalizing the gene expression with the geometric mean of two housekeeping genes (Cyclophillin A and D-glyceraldehyde-3-phosphate dehydrogenase).

### Histological analyses

The micropellets and the biphasic tissues analyzed via histology were first fixed in 4 % paraformaldehyde (PFA, Sigma) for 30 min to preserve the tissue structure, embedded in optimum cutting temperature compound (OCT, Tissue-Tek) and then frozen at −20 °C prior to sectioning. A cryostat (Leica) was used at −25 °C to cut 10-μm-thick sections, which were then placed on poly-lysine slides (Thermo Fischer), dried at room temperature and stored at −20 °C. For staining, the sections were brought to room temperature, and to prevent detachment the sections were fixed a second time onto glass slides with 4 % PFA for 20 min and then rinsed with PBS.

Alcian blue staining was performed to identify the presence of sGAG in the tissues. Sections were covered and incubated for 10 min with 1 % Alcian blue (Sigma) solution in 3 % acetic acid (pH 2.5). Then, the sections were rinsed with PBS and counterstained with 4′,6 diamidino-2-phenylindole (DAPI, Sigma) for 5 min, rinsed with PBS and mounted (CC/mount, Sigma) for imaging.

Alizarin red staining was performed to assess osteogenic matrix deposition. The sections were rinsed with ddH_2_O, dried at 37 °C for 10 min, incubated with Alizarin Red S stain (Sigma) for 10 min, washed with PBS and counterstained with DAPI for 5 min. Finally, the sections were washed with PBS and then mounted for imaging.

For immunofluorescence (IF) analyses, borders were drawn around the sections with a PAP pen (Sigma). A solution of 3 % goat serum (Invitrogen), 0.3 % Triton X-100 in 1 % bovine serum albumin (BSA, Sigma) was used to block sections for 20 min. The sections were incubated in primary antibody dilutions for human Collagen types I, II, or X (raised in mouse, rabbit, and rabbit, respectively; Abcam) overnight at 4 °C in a humidified environment. Negative controls for collagen types I, II and X were treated the same, except the solution did not contain any primary antibody. Next day, the primary antibody solution was washed with 0.3 % Triton X-100 for 3 min twice and then rinsed with PBS once. Subsequently, the corresponding secondary antibody solutions (Cy-3 conjugated anti-rabbit IgG, fluorescein-isothiocyanate-conjugated anti-mouse IgG2b, Abcam) were added to the sections, which were then incubated for 30 min at room temperature, washed with 0.3 % Triton X-100 for 3 min twice, counterstained with DAPI for 5 min, rinsed with PBS and mounted for imaging.

The OsteoImage mineralization assay (Lonza) was used as in the manufacturer’s protocol to stain the inorganic hydroxyapatite component of bone matrix specifically. For the biphasic tissues, the sections were stained and imaged. For micropellet tissues, the whole micropellets were stained and imaged via confocal microscope. All samples were counterstained with DAPI.

### Microscopy

The imaging of sections was performed by using an ECLIPSE Ti epifluorescent microscope (Nikon, Japan) and assessed with NIS Elements BR 3.2 software. Micropellet images and fluorescent histology images were taken with a Nikon DS-Qi1Mc camera, whereas color images (Alcian blue and Alizarin red staining) were recorded by a Nikon DS-Fi1 camera. The 3D images of whole micropellets stained with OsteoImage were acquired by using a confocal microscope (ZEISS, Germany).

### Statistical analyses

The experiments were repeated with three different MSC donor populations. Some differences in differentiation efficiency were observed between the various donors; however, the relationships between the various experimental groups were reproducible and therefore, representative data sets are presented. In each experiment, each condition had *n* = 4 biological replicates. Data are represented in graphs as means + standard deviation (SD). Significance analyses were performed by using SPSS Statistics 21 (IBM, USA) and one-way analysis of variance (ANOVA) with Tukey’s post-hoc tests. For multiple comparisons, a *P*-value smaller than 0.05 was considered as being significantly different. Significance is indicated with Roman numerals, groups with same numerals being statistically similar and groups with different numerals being statistically different.

## Results and discussion

### Flow cytometry characterization of MSC

We used MSC from four different de-identified bone-marrow donations, which were received from a 55-year-old male, a 20-year-old male and a 23-year-old female. Two donations from the same 55-year-old donor were collected at different times. The CD marker expressions for donor MSC at Passage 2 are displayed in Figure S1. All MSC cultures contained populations with <5 % positive for CD45, CD34, or HLA-DR. All MSC cultures contained populations with >95 % positive for CD90, CD73, CD105, CD44 and CD146.

### Chondrogenic and osteogenic features of micropellets

**Fig. 1:**
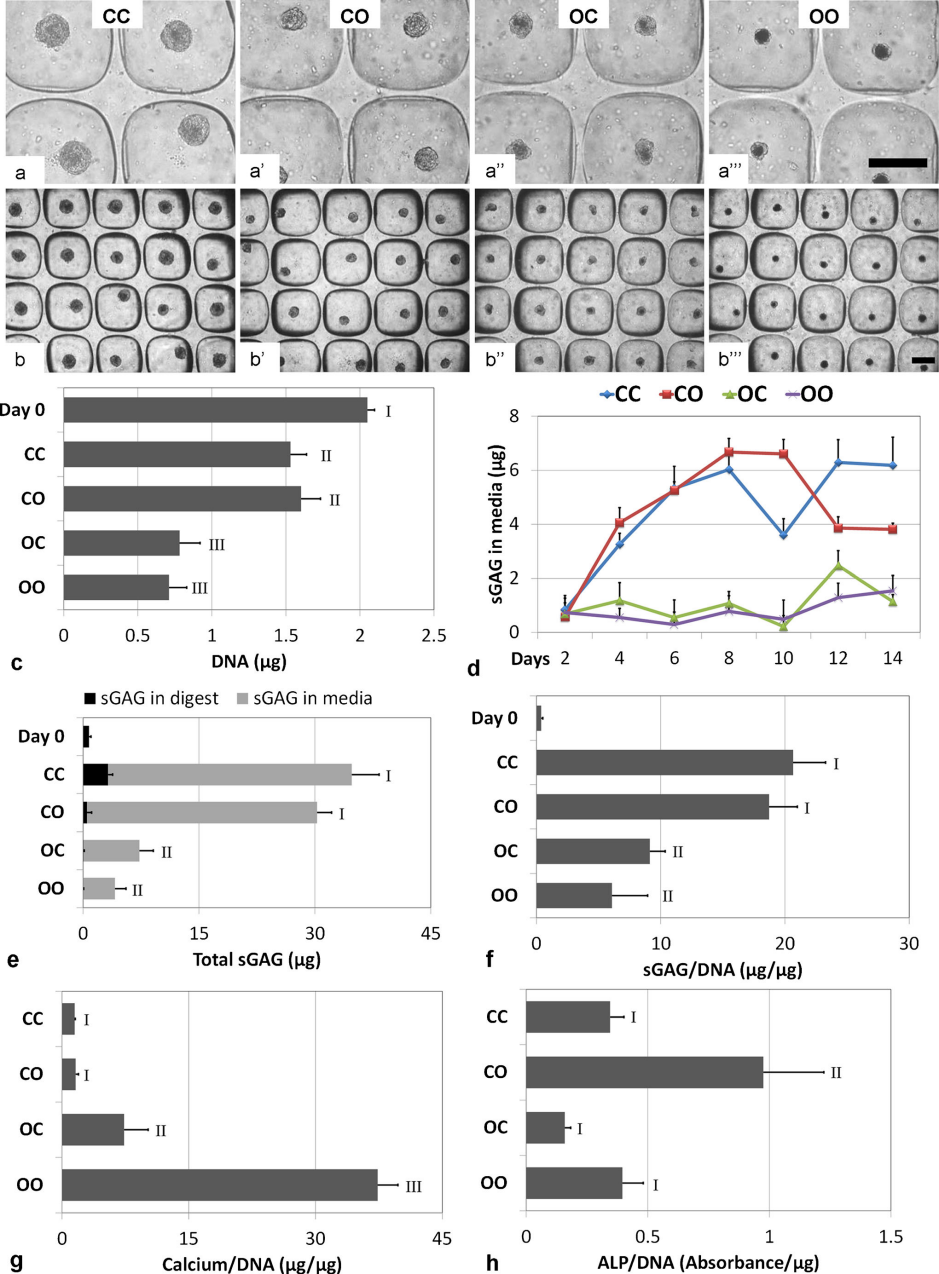
Morphology of micropellets, chondrogenic/osteogenic differentiation and DNA content assessment. **a–a’’’**, **b–b’’’** Size of the micropellets was greatest for CC and smallest for OO micropellets. The core of OO micropellets appeared more opaque. *Bars* 200 μm. **c** Quantity of DNA was lower than day 0 control under all conditions and was significantly reduced for the cultures initiated with osteogenic medium, namely OC and OO. **d** Elution of sulfated glycosaminoglycan (*sGAG*) into the medium was monitored throughout culture; sGAG in medium followed an increasing pattern for micropellets initiated with chondrogenic medium (CC, CO), whereas it was lower and steady for OC and OO micropellets. **e** Quantity of sGAG retained in the tissue at the end of day 14 (*sGAG in digest*) was the greatest for CC micropellets; however, the quantity of total sGAG eluted into the medium (*sGAG in media*) was substantially greater than the quantity retained within the micropellets. **f** Total sGAG/DNA ratio, which is equal to (sGAG in digest + sGAG in media) / DNA, indicated that cultures initiated with chondrogenic medium, namely CC and CO, were able to produce a greater quantity of sGAG during the 2-week culture. **g** Calcium/DNA ratio indicated that OO micropellets had the greatest calcium content. **h** Alkaline phosphatase (*ALP*) activity was significantly greater only in CO micropellets. Data represented in graphs are means + SD, *n* = 4. *Bars* 200 μm

The size of the CC and OO micropellets were noticeably different following 14 days of culture (Fig. 1a-a’’’, b–b’’’): OO micropellets were smaller and had opaque cores. When compared with OO, OC micropellets were larger and had less opaque cores (Fig. 1a–a’’’). DNA quantification revealed that all cultures had less DNA than the day 0 samples. However, the cultures initiated with osteogenic medium, namely OC and OO, had significantly less DNA than the cultures initiated with chondrogenic medium, namely CC and CO (Fig. 1c). DNA content is not the only factor that could have affected micropellet size; the accumulation of a matrix can also vary the size of the micropellets. In this case, however, both the micropellet size and DNA content were lower in the OC and OO micropellets suggesting that greater cell death occurred in those micropellets.

The quantity of sGAG in the micropellets and the culture medium was measured. sGAG quantification is commonly utilized to as an indirect measure of chondrogenesis. Figure 1d was plotted by using the sGAG quantity found in the medium after each medium exchange in order to obtain the elution pattern of sGAG into the medium over the 2-week culture period. The quantity of sGAG in the medium incrementally increased in CC and CO micropellets, while it was lower and constant for OC and OO micropellets over the first 8 days (Fig. 1d). Following day 8, OC and OO micropellets continued to elute only minor quantities of sGAG into the medium. Over the second week of the culture, sGAG elution in CO micropellets tapered slowly at first and then significantly at day 12, because of the switch to the osteogenic medium (Fig. 1d). Figure 1e demonstrates the sGAG found in the tissue on day 14 (sGAG in digest) and the accumulation of sGAG eluted into the medium, which is the summation of sGAG quantities found in the medium after medium exchanges (sGAG in media). The quantity of sGAG retained in the micropellets was the greatest for CC, although the total quantity of sGAG eluted into the culture medium was similar for both the CO and CC micropellet cultures (Fig. 1e). Figure 1f demonstrates the ratio of the total sGAG quantity (which is the summation of sGAG in digest and total sGAG in media) to the corresponding DNA quantity for each sample. The sGAG/DNA ratio was the greatest for CC and CO micropellets and lower for OC and OO micropellets (Fig. 1f). Overall, DNA and sGAG/DNA data indicated that the cultures initiated with a similar medium type over the first 8 days had a similar phenotype following 14 days total culture.

In contrast to chondrogenic characterization, osteogenic characterization provided greater resolution and indicated that the change in medium composition did indeed influence micropellet phenotype substantially. The calcium/DNA ratio was the greatest for OO micropellets, being significantly lower for OC micropellets and was the lowest for CC and CO micropellets (Fig. 1g). Interestingly, the ALP activity/DNA ratio was markedly greater in the CO micropellets (Fig. 1h). ALP activity is considered a key early indicator of osteogenesis being upregulated during early osteogenesis but then diminished as the cells continue to mature (Aubin 2001). The elevated ALP activity observed in the CO micropellets suggests that these tissues acquire a pre-osteogenic phenotype in response to the change from chondrogenic to osteogenic medium.

### Histological features of micropellets

**Fig. 2:**
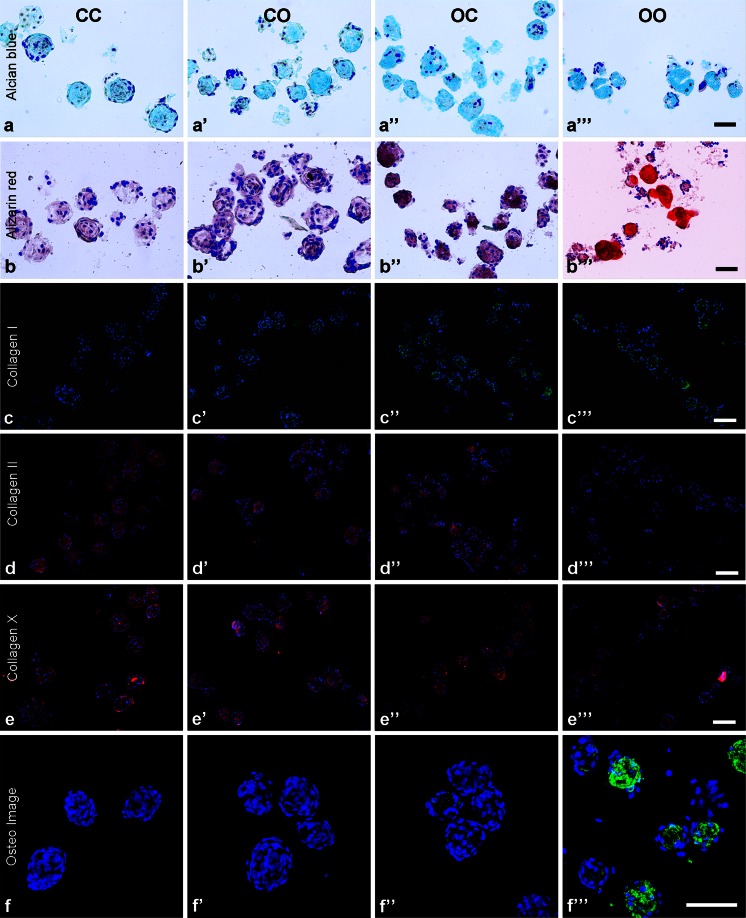
Histological assessment of micropellets. **a–a’’’** Alcian blue staining was similar for all conditions. **b-b’’’** Alizarin red staining indicated that calcification only occurred in OO micropellets. **c-c’’’** collagen I staining was less intense in CC micropellets. **d-d’’’** Accumulation of collagen II was lowest in OO micropellets. **e-e’’’** Collagen X was present in all cultures. **f-f’’’** OsteoImage, specific for hydroxyapatite, intensely stained OO micropellets but only marked hydroxyapatite nucleation points in OC micropellets. *Bars* 200 μm

Alcian blue staining indicated that all micropellets contained sGAG, whereas a lack of Alizarin red staining indicated that no mineralization occurred in CC or CO micropellets (Fig. 2a–a’’’, b-b’’’). Positive Alcian blue staining of OC and OO micropellets probably reflected the expression of sGAG molecules commonly found in the bone (chondroitin sulfate) and that biphasic tissue had been cultured in chondrogenic induction medium for 8 days (Vejlens 1971; Engfeldt and Hjerpe 1976). As expected, Alizarin red staining was the most intense for OO micropellets (Fig. 2b-b’’’). The presence of the mineralized matrix in the OO micropellets was further validated with OsteoImage fluorescent stain, which is specific to the hydroxyapatite portion of the bone matrix (Fig. 2f’’’). OC micropellets also stained positively with OsteoImage, marking small and localized hydroxyapatite nucleation points (Fig. 2f’’) indicating that, when the medium was changed to the chondrogenic type, their osteogenic differentiation was brought to a halt. Collagen II was present in CC, CO and OC micropellets suggesting that chondrogenic medium exposure was required for collagen II accumulation; therefore, OO micropellets were stained less intensely for collagen II (Fig. 2d-d’’’). Collagen X was present in all micropellets, which indicated that MSC micropellets were hypertrophic, regardless of medium type (Fig. 2e-e’’’). Collagen I was present in CO, OC and OO micropellets suggesting that osteogenic medium exposure was necessary to trigger collagen I accumulation; therefore, CC micropellets had less intense collagen I staining (Fig. 2c-c’’’).

### Gene expression in micropellets

**Fig. 3:**
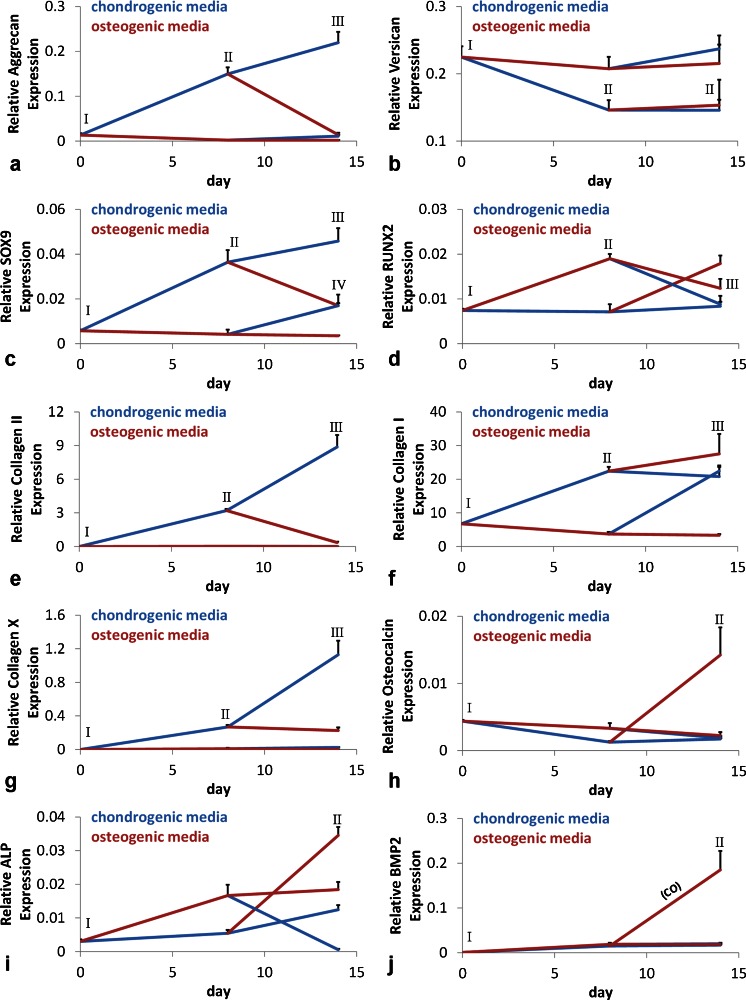
Gene expression analysis of micropellets. Gene expression was assessed at three different time points; day 0, day 8 and day 14. The *lines* connecting time points were colored either *blue* for chondrogenic medium or *red* for osteogenic medium (*continuous blue line* CC, *continuous red line* OO). **a**, **c**, **e**, **g** Aggrecan, SOX9, collagen II and collagen X were upregulated in CC micropellets. **b** Versican was downregulated in chondrogenic-medium-initiated cultures. **d** RUNX2 was greatest in CO micropellets. **f** Collagen I was upregulated in CC, CO and OC micropellets. **h–j** Osteocalcin, alkaline phosphatase (*ALP*) and bone morphogenetic protein 2 (*BMP2*) expressions were significantly greater in CO micropellets at day 14. Data in graphs are given as means + SD, *n* = 4

The gene expression for chondrogenic, hypertrophic and osteogenic markers (aggrecan, SOX9, collagen II and X, versican, RUNX2, collagen I, osteocalcin, ALP and bone morphogenetic protein 2 [BMP2]) were assessed. Aggrecan, SOX9 and collagen II were similarly upregulated at the end of an 8-day chondrogenesis and the expression of those genes was further elevated following 14 days of chondrogenic differentiation (Fig. 3a, c, e). These results indicated that chondrogenesis continued to progress over the full 14 days of chondrogenic culture. SOX9 but not the chondrogenic matrix molecules (aggrecan or collagen II), was upregulated in CO and OC micropellets exposed to chondrogenic differentiation medium for the first 8 days or during the last 6 days, respectively. This suggested that the short-term exposure to chondrogenic medium upregulated SOX9 expression but was insufficient to generate a stable chondrogenic phenotype with high collagen II expression. This paralleled a previous report in which the lack of correlation between SOX9 and collagen II expressions was shown in articular chondrocytes (Aigner et al. 2003). Collagen X expression was upregulated during the first 8-day chondrogenic culture and then remained at a similar level in CO micropellets but increased in CC micropellets (Fig. 3g). Versican expression was significantly downregulated in micropellets initiated with chondrogenic medium, namely CC and CO, when compared with day 0. By contrast, versican expression was similar to that at day 0 at the end of culture for tissues initiated in osteogenic medium, namely OC and OO (Fig. 3b). The expression of collagen I was elevated with chondrogenic medium supplementation (Fig. 3f). This pattern suggested that exposure to chondrogenic medium in the culture directed collagen I expression. TGF-β1 contained in the chondrogenic medium is known to upregulate collagen I expression specifically during development in various tissues, including bone (Sandberg et al. 1988; Ignotz et al. 1987; Heine et al. 1990). RUNX2 was upregulated in CO and OO conditions (Fig. 3d). Osteocalcin, ALP and BMP2 expressions exhibited a similar pattern, being significantly higher in CO micropellets than in the others (Fig. 3h–j). This finding is interesting because, despite the lack of calcification in CO tissues when compared with OO, ALP activity and also gene expression for osteogenic factors such as ALP, BMP2, osteocalcin and RUNX2 were significantly elevated in CO micropellets. This parallels recent findings (Farrell et al. 2011; Mueller et al. 2010; Scotti et al. 2010) and suggests that pre-conditioning MSC with chondrogenic medium prior to osteogenic differentiation enhances bone formation by better mimicking the natural process of endochondral ossification.

### Biphasic tissue assembly and histology

Results from the first part of our study indicated that an 8-day differentiation cycle was insufficient for MSC micropellets to acquire the chondrogenic and osteogenic phenotypes required for use in the assembly of a biphasic tissue. Specifically, whereas the OC micropellets did not demonstrate chondrogenic features after the medium was swapped, they also did not achieve a similar state of tissue mineralization as did the OO micropellets. This implies that the osteogenic phenotype was not acquired after only 8 days in osteogenic induction medium. Similarly, the CO micropellets showed chondrogenic features after the first 8 days of culture; however, when the medium was altered from chondrogenic to osteogenic, they rapidly became pre-osteogenic, parallel to previous findings (Farrell et al. 2011; Mueller et al. 2010; Scotti et al. 2010). This result implies that the chondrogenic phenotype is particularly unstable and that exposure of early-stage chondrogenic micropellets to an osteogenic medium would result in an osteogenic phenotype. Based on these observations, we reasoned that the separate differentiation cultures would need to be maintained for 14 days before assembling the micropellets into a biphasic tissue cultured in a common medium formulation.

**Fig. 4:**
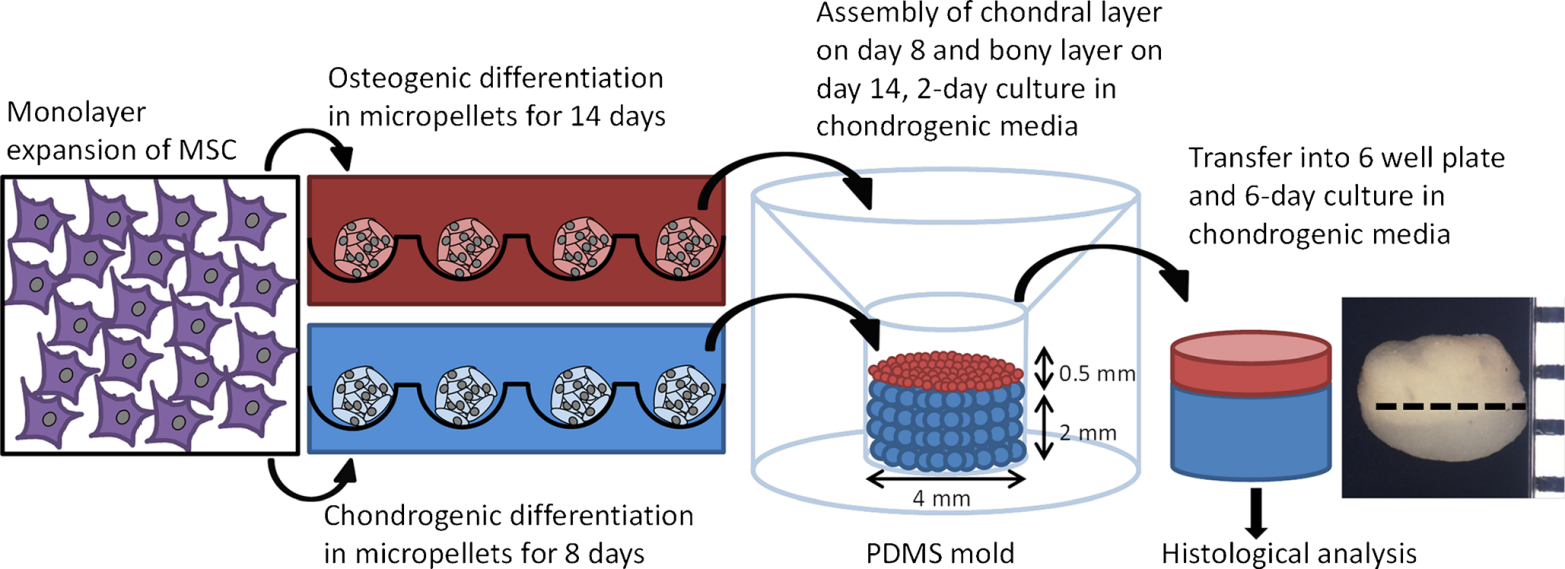
Biphasic tissue construction with micropellets. The monolayer-expanded mesenchymal stem/stromal cells (*MSC*) were used to form micropellets and cultured in chondrogenic medium and osteogenic medium separately. The chondrogenic micropellets were assembled on day 8 and then the osteogenic micropellets were layered on top at day 14 by using a custom-made polydimethylsiloxane (*PDMS*) mold. The biphasic tissue was cultured for another week and then sectioned for histological analysis

Our own work in this area indicates that the first phase of discrete culture cannot be drawn out indefinitely, as the capacity of cartilage micropellets to amalgamate is reduced as the tissues mature and the matrix accumulates (Babur et al. 2013). Similarly, more efficient fusion of human MSC conventional pellets at earlier days of chondrogenic culture has been shown (Bhumiratana et al. 2014). Taking these observations together, we elected to initiate the chondrogenic and osteogenic micropellet differentiation cultures on the same day (day 0), assemble the chondrogenic micropellets into a cartilage-only tissue at day 8 in chondrogenic induction medium, then layer the osteogenic micropellets onto the cartilage tissue at day 14 and continue the biphasic tissue culture in chondrogenic induction medium for an additional week (Fig. 4).

The PDMS mold used for osteochondral tissue formation (Fig. S2) had a diameter of 4 mm and a total of 12,000 chondrogenic micropellets filled the mold to a height of 2 mm and the same number of smaller osteogenic micropellets filled it to an additional 0.5 mm in height (Fig. 4). As the biphasic tissue matured, the total diameter decreased slightly to ∼3 mm, although the chondrogenic layer remained ∼2 mm deep and the osteogenic layer shrunk slightly to ∼0.3 mm deep. The shrinkage in tissue size and deviation from a perfect cylinder in tissue morphology (Fig. 4) might have been caused by the MSC aggregate “compaction” phenomenon (Sart et al. 2014).

**Fig. 5:**
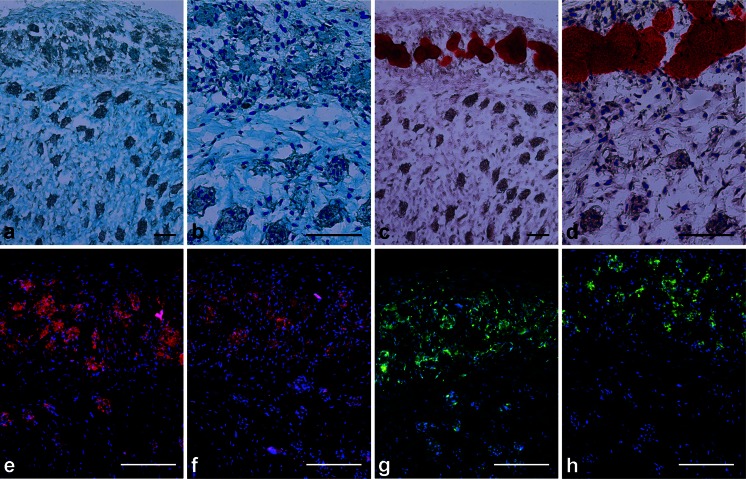
Histological assessment of osteochondral-like tissue. The *upper part* of the tissue is the bony layer and the *lower part* is the chondral layer. **a**, **b** Alcian blue stains both the chondral and bony layers. **c**, **d** Alizarin red specifically stains the calcified region in the bony layer. **b**, **d** At higher magnification, the cells migrating out of the individual micropellets and filling the void space between micropellets are visible. **e**, **f**, **g** Collagens II, X, and I were all accumulated more on the bony side, although being present on both sides. **h** OsteoImage stains exclusively the bony side indicating the lack of calcification in the chondral layer. *Bars* 200 μm

The cross-sectional area of the osteochondral tissue was assessed for histological features of bone and cartilage. Alcian blue stained the tissue throughout (Fig. 5a, b). This staining highlighted individual micropellets, although discrete micropellets were much more visible in the cartilage portion of the tissue (Fig. 5a, b). By contrast, the Alizarin red exclusively stained the bony side of the tissue; however, staining revealed that mineralization was not always continuous (Fig. 5c, d). In higher magnification images, individual cells clearly bridged the two tissue layers (Fig. 5b, d). Unexpectedly, the collagen II staining was more intense in the bony layer relative to the chondral layer (Fig. 5e). This differed from the first part of our study in which the micropellets were cultured for 14 days in discreet microwells and exhibited minimal collagen II expression in the OC medium combination (Fig. 3e). This difference suggests that the co-culture of the osteogenic and chondrogenic tissues drives this outcome. One possible explanation is that chondrogenically induced cells from the cartilage micropellets migrated onto the osteogenic-induced micropellets, forming a layer of cells that contributed to the observed intense collagen II staining. An alternative possibility is that paracrine signals from the adjacent cartilage tissues modified the behavior of the initially osteogenically induced cells, resulting in their increased expression of collagen II. A precedent exists for a change in cell behavior in response to the fusion of pellets and for the signaling between adjacent fused pellets (Lehmann et al. 2013). For example, Lehmann et al. (2013) provided high-resolution images demonstrating that the cells that form the layer that fuses the pellets together contribute to a unique tissue layer with histological properties that differ from the pellets that were fused to generate the amalgamated tissue. Further experiments will be required to elucidate the mechanism that drives the enhanced collagen II staining observed in our osteogenic tissue layer.

Collagen X (Fig. 5f) and I (Fig. 5g) staining was present in both layers but was more intense in the bony layer, revealing a lower collagen content in the chondral layer; this was consistent with our expectations for bone-like tissue (Farrell et al. 2011; Mueller et al. 2010; Scotti et al. 2010). The hydroxyapatite-specific OsteoImage exclusively stained the bony layer suggesting no accumulation of undesirable mineralization within the chondral layer (Fig. 5h). Our work builds on previous work demonstrating that discreet cartilage microtissues can be fused together to form a cartilage-like core with a bone-like exterior (El-Serafi et al. 2011). Specifically, our work demonstrates the possiblity of assembling cartilage-like micropellets and bone-like micropellets to make a structured bi-phasic tissue. We believe that this capacity to assemble a complex layered tissue will contribute to future efforts to repair or regenerate articular cartilage.

### Concluding remarks

Herein, we described methods for the manufacture of osteogenic and chondrogenic micropellets as building blocks from bone marrow-derived MSC. Using the micropellet approach, we first assessed the impact of various medium conditions on osteogenic and chondrogenic differentiation of MSC. Then a scaffold-free osteochondral tissue was assembled via layering osteogenic and chondrogenic micropellets in a tailored culture device. The micropellets fused into a continuous tissue retaining the two distinct original layers. Previously, the generation of an osteochondral tissue interface by using collagen microencapsulated rabbit MSC spheroids has been described (Cheng et al. 2011). However, to our knowledge, this is the first scaffold-free biphasic tissue built with a single cell type and our results are the first to demonstrate that a continuous bi-phasic structure can be generated without the need for a hydrogel or polymer support structure. In future studies, the use of micropellets should enable the precise layering of various micropellets and the more accurate replication of the complex zonal structure found in native cartilage.

## Electronic supplementary material

Table S1 Primers used for gene expression analysis. (DOCX 14 kb)

Figure S1 Flow cytometry characterization of MSC. The MSC from all three donors used in these studies are characterized for their expression of CD45, CD34, HLA-DR, CD90, CD73, CD105, CD44 and CD146. (GIF 148 kb)

High resolution image (TIFF 1363 kb)

Figure S2 Generation of PDMS mold used to assemble micropellets. The tip of a 10 mL syringe was cut and glued to the middle of a single well in a 6-well plate and the space between the syringe and the well was filled with PDMS and cured. Then the syringe tip was removed and the extra PDMS layer around the mold was cut out to accommodate more medium in the same well. (GIF 31 kb)

High resolution image (TIFF 68 kb)

Figure S3 Negative controls for collagen antibody staining in biphasic tissue. Negative control (no primary antibody) for collagen II (**a**), collagen X (**b**) and collagen I (**c**). (GIF 101 kb)

High resolution image (TIFF 1325 kb)
