# Supplementary material for: High-throughput bone and cartilage micropellet manufacture, followed by assembly of micropellets into biphasic osteochondral tissue
Source: Cell Tissue Res. 2015 Apr 30;361(3):755–68. doi: 10.1007/s00441-015-2159-y (PMC4550660; doi:10.1007/s00441-015-2159-y)
Supplement: Supplementary file 1 — Primers used for gene expression analysis. (DOCX 14 kb) [file 441_2015_2159_MOESM1_ESM.docx]

**Table S1**

| **Gene (Amplicon size in basepair)** | **Primers** |
| --- | --- |
|  |  |
| Cyclophilin A (164) | Forward CTCGAATAAGTTTGACTTGTGTT |
|  | Reverse CTAGGCATGGGAGGGAACA |
| GAPDH (119) | Forward ATGGGGAAGGTGAAGGTCG |
|  | Reverse TAAAAGCAGCCCTGGTGACC |
| SOX9 (77) | Forward TTCCGCGACGTGGACAT |
|  | Reverse TCAAACTCGTTGACATCGAAGGT |
| Aggrecan (85) | Forward TCGAGGACAGCGAGGCC |
|  | Reverse TCGAGGGTGTAGCGTGTAGAGA |
| Collagen II, COL2A1 (79) | Forward GGCAATAGCAGGTTCACTGACA |
|  | Reverse CGATAACAGTCTTGCCCCACTT |
| Collagen I, COL1A1 (83) | Forward CAGCCGCTTCACCTACAGC |
|  | Reverse TTTTGTATTCAATCACTGTCTTGCC |
| Versican (98) | Forward TGGAATGATGTTCCCTGCAA |
|  | Reverse AAGGTCTTGGCATTTTCTACAAAG |
| Collagen X, COL10A1 (70) | Forward CAAGGCACCATCTCCAGGAA |
|  | Reverse AAAGGGTATTTGTGGCAGCATATT |
| Runx2 (113) | Forward GGAGTGGACGAGGCAAGAGTTT |
|  | Reverse AGCTTCTGTCTGTGCCTTCTGG |
| Osteocalcin (70) | Forward GAAGCCCAGCGGTGCA |
|  | Reverse CACTACCTCGCTGCCCTCC |
| ALP (89) | Forward CGTGGCTAAGAATGTCATCATGTT |
|  | Reverse TGGTGGAGCTGACCCTTGA |
| BMP2 (72) | Forward AAAACGTCAAGCCAAACACAAA |
|  | Reverse GTCCACGTACAAAGGGTCTCTCT |
