# Supplementary figures and images for: High-throughput bone and cartilage micropellet manufacture, followed by assembly of micropellets into biphasic osteochondral tissue
Source: Cell Tissue Res. 2015 Apr 30;361(3):755–68. doi: 10.1007/s00441-015-2159-y (PMC4550660; doi:10.1007/s00441-015-2159-y)

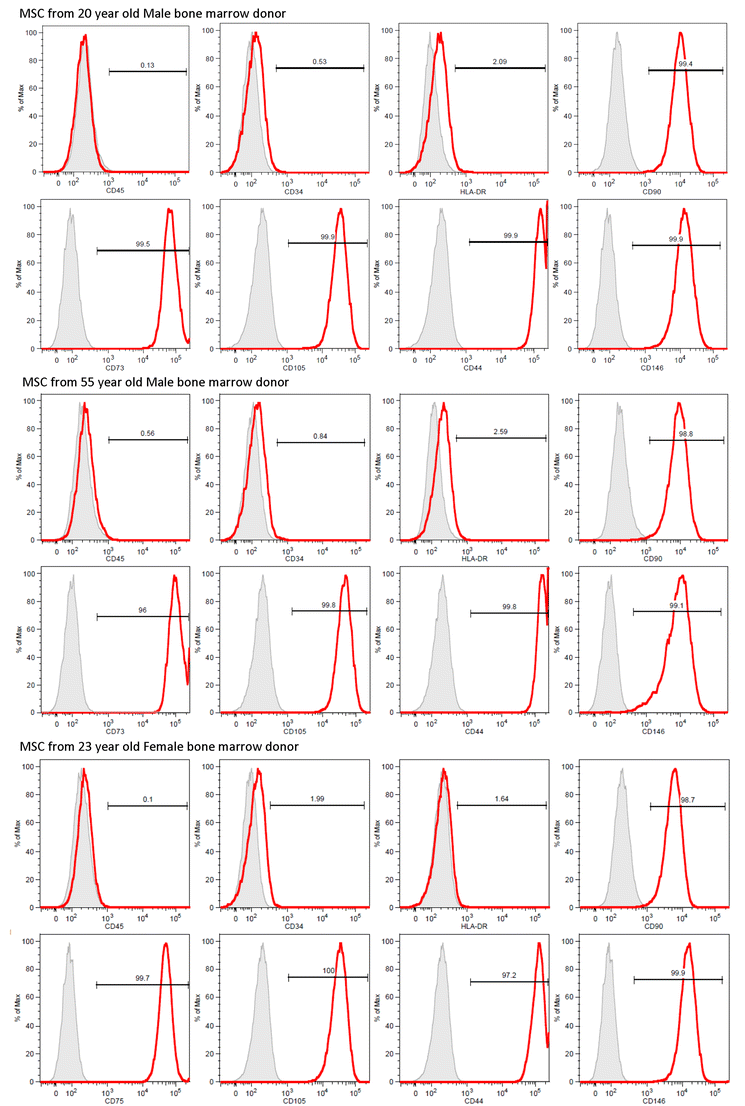

Supplement: Supplementary file 2 — Flow cytometry characterization of MSC. The MSC from all three donors used in these studies are characterized for their expression of CD45, CD34, HLA-DR, CD90, CD73, CD105, CD44 and CD146. (GIF 148 kb) [file 441_2015_2159_Fig6_ESM.gif]

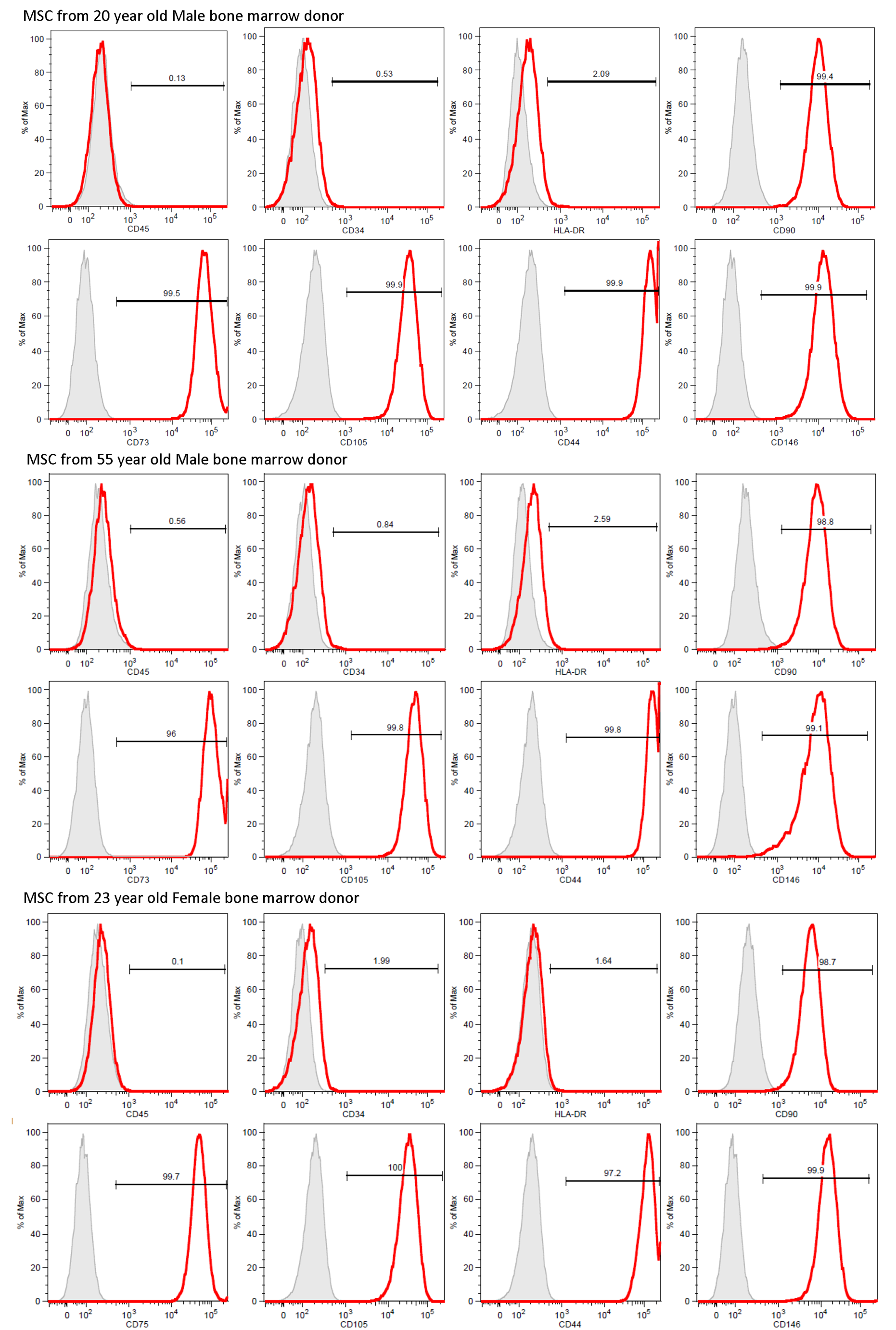

Supplement: Supplementary file 3 — High resolution image (TIFF 1363 kb) [file 441_2015_2159_MOESM2_ESM.tif]

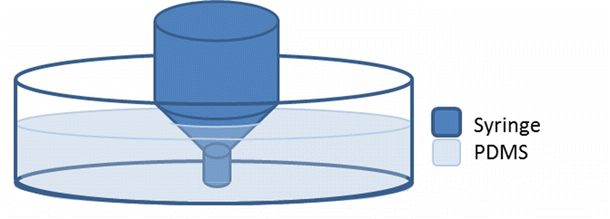

Supplement: Supplementary file 4 — Generation of PDMS mold used to assemble micropellets. The tip of a 10 mL syringe was cut and glued to the middle of a single well in a 6-well plate and the space between the syringe and the well was filled with PDMS and cured. Then the syringe tip was removed and the extra PDMS layer around the mold was cut out to accommodate more medium in the same well. (GIF 31 kb) [file 441_2015_2159_Fig7_ESM.gif]

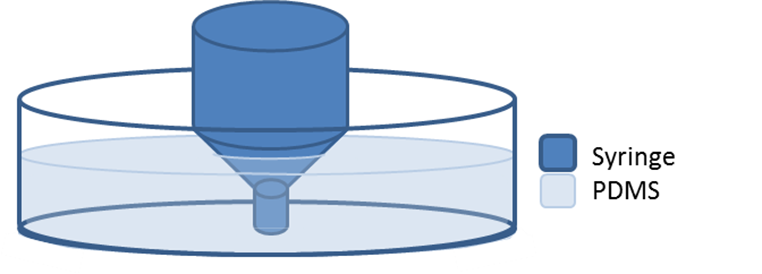

Supplement: Supplementary file 5 — High resolution image (TIFF 68 kb) [file 441_2015_2159_MOESM3_ESM.tif]

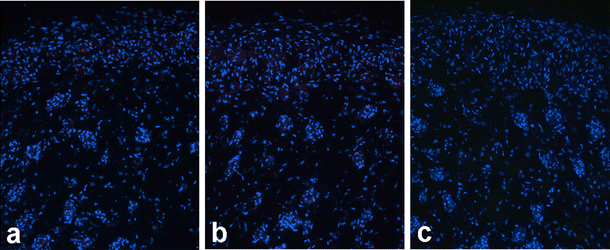

Supplement: Supplementary file 6 — Negative controls for collagen antibody staining in biphasic tissue. Negative control (no primary antibody) for collagen II (a), collagen X (b) and collagen I (c). (GIF 101 kb) [file 441_2015_2159_Fig8_ESM.gif]

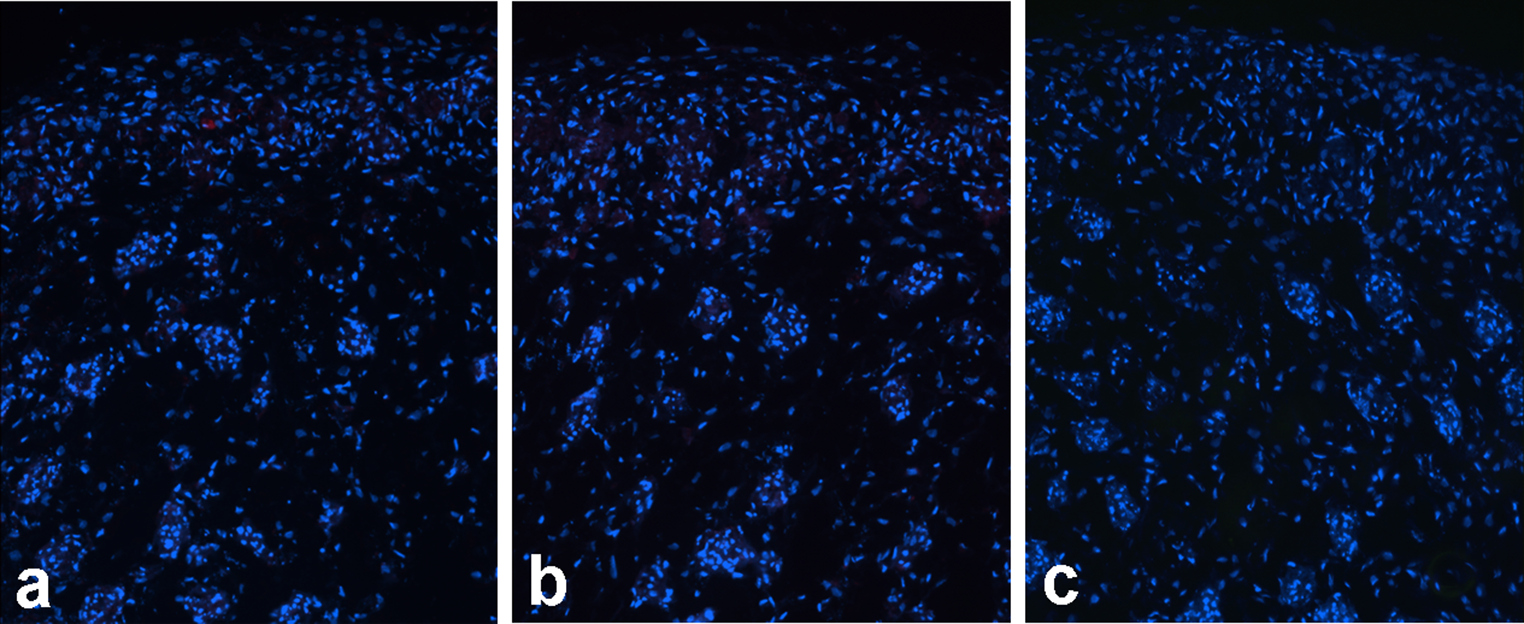

Supplement: Supplementary file 7 — High resolution image (TIFF 1325 kb) [file 441_2015_2159_MOESM4_ESM.tif]
